# Is health-related quality of life associated with adequacy of hemodialysis in chronic kidney disease patients?

**DOI:** 10.1186/s12882-021-02539-z

**Published:** 2021-10-07

**Authors:** Lamia M. Hasan, Dina A. H. Shaheen, Ghada A. H. El Kannishy, Nagy A. H. Sayed-Ahmed, Ahmed M. Abd El Wahab

**Affiliations:** 1 Mansoura Nephrology and Dialysis Unit (MNDU), Mansoura University Hospital, Mansoura, Egypt; 2 Rheumatology and Immunology Unit, Internal Medicine Department, Mansoura University Hospital, Mansoura, Egypt

**Keywords:** Hemodialysis, HRQoL, Dialysis adequacy, KDQOL-SF

## Abstract

**Introduction:**

Monitoring Health Related Quality of Life (HRQoL) in different stages of chronic kidney disease is advised by all nephrology societies. We aimed to study the relation between quality of life and dialysis adequacy.

**Methods:**

One hundred patients (51% males), on regular hemodialysis 3/week for > 6 months in two hospitals were included. Single pool Kt/V was used to assess dialysis adequacy. Patients were grouped into 3 divisions according to Kt/v: Group A > 1.5 (*n* = 24), group B 1.2–1.5 (*n* = 54) and group C < 1.2 (*n* = 22). KDQOL-SF™ questionnaire was used to study quality of life in our groups. Group C was reassessed after 3 months of improving Kt/v.

**Results:**

Mean values were: Kt/V (1.48 ± 0.41), Cognitive Function (84.27 ± 9.96), Work status (30.00 ± 33.33), Energy (45.70 ± 13.89), Physical Function and Role limitations due to physical function (45.30 ± 12.39 and 31.25 ± 19.26, respectively). *Group A had significantly higher* scores of *KDQOL-SF except* Role limitations due to Physical Function. All subscales improved in group C after Kt/v improvement except *3 subscales, namely,* work status, patient satisfaction and role limitation due to physical and emotional functions.

**Conclusion:**

Inadequate HD badly affects quality of life and improving adequacy refines many components of quality of life.

## Introduction

End stage renal disease (ESRD) is an increasing issue worldwide impairing patients Qol [1]. Hemodialysis (HD) is the mostly used for treatment [2] leading to overt changes in patients’ lives [3, 4].

Improving dialysis adequacy, anemia, hyperparathyroidism and many other factors is known to lessen morbidity and mortality in dialysis patients [5–8].

Dialysis adequacy evaluation is difficult as many factors like volume status, electrolytes and acid base interfere with it but Kt/v is the mostly used parameter.

The reported prevalence of ESRD in Egypt is estimated to be 624 pmp [9].

Many physicians looking after hemodialysis patients are interested mainly in the clinical and laboratory data of their patients, paying little attention to the patients’ emotional satisfaction and their quality of life.

Most of regular HD patients are vulnerable for many physical and psychological disabilities like easy fatigability, myalgia, depression and sexual dysfunction [10].

Many disease-specific questionnaires such as the World Health Organization Quality of Life Survey (WHOQoL), Short Form (SF)-36 health questionnaire and the Choices Health Experiences Questionnaire [CHEQ] are used to assess HRQoL in ESRD [11].

Of the 36 items in the SF-36 questionnaire, we only used 35 items representing 2 summary measures, the physical component summary (PCS), mental component summary (MCS) and 8 scales [12].

Advantages of KDQOL-SF are: 1) Tested in different populations with renal disease. 2) It includes general and specific modules evaluating chronic kidney disease. 3) Effective in hemodialysis and peritoneal dialysis patients’ assessment. 4) It can be self-applied or applied by an interviewer. 5) It has been culturally adapted and validated in various languages [13].

This work was planned to focus on assessing HRQOL among ESRD patients treated with hemodialysis in relation to different variables, especially the adequacy of dialysis relying on the calculated kt/V.

## Patients and methods

### Study

This study was conducted in Mansoura Nephrology and Dialysis Unit (MNDU) and Talkha hemodialysis units from 2017 to 2018.

Criteria of inclusion:

- Gender: Both
- Age: Older than 20 years
- Maintained on HD (thrice weekly) for ≥6 months.
- Patients with controlled DM or hypertension (HTN) (Uncontrolled blood pressure > 150/90 in average weekly predialysis reading while the patient is on maximum doses of 3 or more antihypertensive medications with ideal dry body weight. Uncontrolled DM means FPG > 130 and PPG > 150 in average weekly reading using bedside glucose measuring devices).

The exclusion criteria included:

- Recently dialyzed patients (less than 6 months)
- Patients with cognitive impairment
- Patients with mental retardation
- Patients with COPD
- Patients with severe anaemia
- Patient with uncontrolled DM or HTN
- Patients with other system failure

The study was approved by our faculty IRB, The nature and intent of the study were fully explained to all subjects, and an informed written consent was obtained from each participant before running the study.

One hundred thirty-four patients were screened and 119 patients met the inclusion criteria. One hundred patients out of 119 agreed to participate.

All patients were being subjected to history taking and full examination.

### Study design

The 100 subjects who agreed to participate in this study were divided according to their kt/v into three groups:

- The first group (group A) with kt/v > 1.5 (*n* = 24).
- The second group (group B) with kt/v range from [1.5–1.2] (*n* = 54).
- The third group (group c) with kt/v < 1.2 (*n* = 22).

Measures to improve kt/v were applied to Group C and then all the collected parameters were tested again in this group.

Improving kt/v is done for group C for 3 months through:

1. Adjustment of dialyser surface area
2. Increasing blood flow rates.
3. Extending session time.

### Anthropometric measurements

The patients’ weight in kilograms before and after dialysis sessions, dialysis session duration, ultrafiltration volume, was collected for all patients.

### Specimen collection

Pre-dialysis blood samples were withdrawn into 2 vaccutainer tubes from all subjects at the start of the study and after 3 months for group C. Both predialysis and postdialysis urea samples were withdrawn before and after the same session of HD. Daugirdas et al. [14] formula was used for Calculation of Kt/V:

Kt/V = −ln (Ratio-0.03) + [(4-(3.5 × Ratio)) × (UF/Wt)], in which Ratio is post BUN/pre BUN, UF is ultrafiltration volume, and Wt is post dialysis weight.

Biochemical analysis:

- Complete blood count, Liver function tests, serum calcium, serum phosphorus and iPTH were measured.

### Subjective methods

HRQOL was assessed with the validated Kidney Disease Quality of Life-Short Form (KDQOL-SF version 1.3): http://gim.med.ucla.edu/kdqol/downloads/-download.html [15].

KDQOL-SF 36 issued by Hays et al. in 1994 and is published for free on internet website: http://gim.med.ucla.edu/kdqol/downloads-download.html.

For purpose of simplicity, the KDQOL-SF version 1.3 was summarized in three categories; namely, kidney disease targeted scales, physical component and mental component summaries, each of which drives its core information and calculations from one or a group of questions from the original 36 questions. The user manual of this questionnaire with these details was previously published [16].

The KDQOL-SF can be split in a generic part and a disease-specific part. First, the generic part is formed by the SF-36 version 1. The domains of the SF-36 can be summarized in two summary scores, one for physical functioning (physical component summary—PCS) and one for mental functioning (mental component summary—MCS). These summaries are constructed so that a score of 50 represents the mean of the general United States population with a standard deviation of 10 [16]. Second, the disease-specific part of the KDQOL-SF consists of 44 kidney disease-targeted questions. The responses to these items are condensed in 12 domains. These domains have a score from 0 to 100, with higher scores indicating the absence of problems.

Results from the SF-36 instrument are further summarized into a physical composite summary (PCS) score and a mental composite summary (MCS) score. PCS aggregates items from physical function, role physical, pain, vitality and general health. MCS aggregates items from role emotional, emotional wellbeing, energy, social function and general health. These summaries are constructed so that a score of 50 represents the mean of the general United States population with a standard deviation of 10 [16].

There are some explanations to some questionnaire items:

1. Physical functioning (PF) – the level of limitation of physical activity caused by health limitations
2. Role physical (RP) – helps measure the limitations of patient-specific physical activity caused by health problems
3. Vitality (VT) – measurement of energy and fatigue
4. Social functioning (SF) – defines the level of social life limitations caused by physical and emotional discomfort
5. Mental health (MH)– defines the level of psychological stress and well-being [17].

A previous study, carried out in Alexandria, Egypt [18], utilized an Arabic translation of the KDQOL-SF version 1.3 and the authors concluded that this translation is a validated and reliable tool for studying the HRQOL in ESRD patients. In the current study, this Arabic version of KDQOL-SF1.3 was also used for assessment of HRQOL with some additional section translated from the original English version to suit the patients on regular hemodialysis. The questionnaire was used to assess the studied population and to group C before and after improving kt/v. One item studying sexual activity in the KDQOL-SF™ was excluded from the study as nearly all patients refused to talk about that issue (cultural concerns).

#### Scoring system

The standard scoring program of the KDQOL-SF™ 1.3 is based on the Microsoft Excel 97 spreadsheet program and includes information about the computation method:

https://www.rand.org/health-care/surveys_tools/kdqol.html.

The scores for each dimension range from 0 to 100, with higher scores reflecting better HRQOL. The change in health (question 2) of the SF36 scale and the 0–10 overall health rating (question 22) items are scored as single items [13].

### Statistical methods

Data were tabulated, coded then analyzed using the computer program SPSS (Statistical package for social science) version 20.0 to obtain.

Descriptive data:

Descriptive statistics were calculated in the form of:

1. Mean ± Standard deviation (SD).
2. Median & interquartile range (IQR) [25, 75].
3. Frequency (Number-percent).

#### Analytical statistics

- In the statistical comparison between the different groups the normality of distribution in continuous data was checked by kolmogorov-smirnov test, and Shapiro-Wilk normality test
- The significance of difference in groups (A, B, C) was tested using Kruscalwalis’ H test
- The comparison between the three groups was done using Bonferroni post-hoc test. The level of significance was set at *p* <  0.05.
- Measurements within the same group C before and after improving, were statistically compared using paired t-test or Wilcoxon signed-ranks test for parametric or non-parametric data, respectively. Results are presented as counts for nominal variables, and as mean ± standard deviation (SD) and median (interquartile range) for continuous variables. The level of significance was set at *p* <  0.05.

## Results

This study involved 100 HD patients with an age ranging between 28 and 62 years (mean 48.8 ± 5.89 years). DM was present in 26% of the patients while HTN was present in 82%. HTN was the cause of ESRD in 58% of the patients while DM was the cause in 22% (Table 1). It included 51 males and 49 females with their weights ranging between 54 and 104 Kg (mean 79.01 ± 11.35 Kg). All the patients were treated with regular HD sessions three times weekly with a median (IQR) duration of 4 h and the interdialytic weight gain ranging between 1 and 4 kg (mean2.60 ± 0.96) per session. The mean kt/v was 1.48 ± 41. As regard the KDQOL_SF v 1.3 scoring system, the mean of the scores of the item related to “Symptoms and Problem” list of the total studied population was 71.29 ± 17.27; a figure that is not far from a maximum possible score of 100 for the least symptoms and problems. The mean of the scores of the item dealing with “Effect of kidney disease” was also reasonable, 65.13 ± 11.03. On the other hand, the mean of the scores of the item dealing with “Work status”, “Energy”, “Physical Function” as well as “Role limitations due to physical function” were very low (30.00 ± 33.33, 45.70 ± 13.89, 45.30 ± 12.39 and 31.25 ± 19.26; respectively), while the mean of the scores of the item referring to “Cognitive Function” was high [mean 84.27 with SD 9.96] (Table 2). Twenty four patients of the studied population had kt/v > 1.5 (group A), 54 patients had kt/v between (1.2 and 1.5; group B) and 22 had Kt/v < 1.2 (group C). The studied general and laboratory variables of the three Kt/v groups (Table 3) were comparable regarding age, pre-dialysis urea and albumin. However, the body weights before sessions were significantly higher in group (A). There was also a highly significant difference between the interdialytic weight gain and session durations between the three groups with the greatest volumes and longest sessions in group (A). On the other hand, calcium and hemoglobin were significantly higher in group A, while PTH and phosphorus were significantly higher in group C versus other groups (Table 3). Regarding the KDQOL-SF V 1.3 questionnaire, the comparison of the scores of the items between the three studied groups, showed highly significant differences between the three groups except for 2 subscales; the “Role limitations due to Physical and emotional function” (Table 4).

**Table 1:** General descriptive characteristics of the total studied patients (*n* = 100)

Age (Year, Mean ± SD)	48.8 ± 5.89
DM (N, %)	26 (26%)
HTN (N, %)	82 (82%)
Original kidney disease	
• HTN (N, %)	58 (58%)
• DM (N, %)	22 (22%)
• Stones, pyelonephritis	12 (12%)
• Undetermined	8 (8%)

**Table 2:** Descriptives of the response to the questionnaire of the total studied patients (*n* = 100)

Questionnaire’s items	Mean ± SD	Median (IQR)
Symptoms / problems list	71.29 ± 17.27	77.08 (70.83–81.25)
Effect of kidney disease	65.13 ± 11.03	65.63 (95.37–71.87)
Burden of kidney disease	55.75 ± 15.78	62.50 (50–68.75)
Work Status	30.00 ± 33.33	25 (0.00–50.00)
Cognitive function	84.27 ± 9.96	86.67 (80.00–93.33))
Quality of Social interaction	64.27 ± 14.63	66.67 (53.33–73.33)
Sleep	64.25 ± 18.34	70 (58.13–75)
Overall health	65.50 ± 20.76	70.00 (70–80)
Patient satisfaction	44.33 ± 11.42	50.00 (33.33–50)
Physical function	45.30 ± 12.39	45.00 (35.0–53.75)
Role limitation due to physical function	31.25 ± 19.26	25.00 (25.0–50)
Pain	49.90 ± 20.67	55.00 (36.87–67.50)
General health	52.40 ± 11.84	50.00 (45–60)
Emotional Well Being	61.36 ± 14.17	68.00 (56.00–72)
Role limitations due to emotional problems	52.67 ± 28.10	66.67 (33.33–66.66)
Social function	54.20 ± 21.84	62.5(50.00–75.00)
Energy	45.70 ± 13.89	50.00 (40.00–55.00))
Physical composite	36.60 ± 5.51	36.70 (32.47–40.20)
Mental composite	42.11 ± 9.11	45.20 (36.11–48.23)

**Table 3:** Comparison between the three groups of patient regarding the general data (*n* = 100)

		Group A	Group B	Group C	*P* value.	Group A vs Group B	Group A vs Group C	Group B vs Group C
Age (Year)	N	24	54	22	0.636			
	Mean	50.45 ± 1.77	49.89 ± 2.38	50.64 ± 3.84				
	Mean Ranks	53.79	47.98	53.09				
Pre-urea (mg/dl)	N	24	54	22	0.206			
	Mean	120.33 ± 28.80	129.93 ± 26.35	129.00 ± 35.17				
	Mean Ranks	41.44	53.94	51.95				
Pre session weight (Kg)	N	24	54	22	0.042	0.036	0.389	1.000
	Mean	83.48 ± 10.54	76.99 ± 11.10	79.09 ± 13.99				
	Mean Ranks	62.98	45.11	50.11				
Interdialytic weight gain (Kg)	N	24	54	22	0.002	1.000	0.006	0.003
	Mean	2.35 ± 1.03	2.42 ± 0 .95	3.20 ± 0.59				
	Mean Ranks	43.44	45.90	69.50				
Dialysis session time (hrs)	N	24	54	22	< 0.0001	0.938	< 0.0001	< 0.0001
	Mean	4.00 ± 0.00	3.89 ± 0.32	3.05 ± 0.043				
	Mean Ranks	63.50	58.00	17.91				
Serum calcium (mg/dl)	N	24	54	22	0.001	0.010	0.001	0.568
	Mean	9.84 ± 0.83	9.22 ± 0.79	8.93 ± 0.88				
	Mean Ranks	68.50	47.59	38.00				
Serum Ph (mg/dl)	N	24	54	21	0.007	1.000	0.013	0.015
	Mean	4.65 ± 0.57	4.68 ± 1.08	5.38 ± 0.77				
	Mean Ranks	42.73	46.56	67.17				
Serum albumin (g/dl)	N	24	54	22	0.704			
	Mean	3.35 ± 0.40	3.27 ± 0.39	3.28 ± 0.46				
	Mean Ranks	54.79	49.00	49.50				
Serum PTH (pg/ml)	N	24	53	22	< 0.0001	0.480	< 0.0001	< 0.0001
	Mean	158.10 ± 150.73	318.89 ± 486.23	750.00 ± 523.20				
	Mean Ranks	35.25	45.18	77.70				
Hb (g/dl)	N	24	54	22	< 0.0001	0.012	< 0.0001	0.059
	Mean	10.20 ± 1.05	9.71 ± 0.86	9.10 ± 0.78				
	Mean Ranks	69.81	49.34	32.27				
Kt/v	N	24	54	22	< 0.0001	< 0.0001	< 0.0001	< 0.0001
	Mean Ranks	88.50	49.50	11.50				

*P* value is calculated by Kruskal-Wallis H test

Comparison between three groups is calculated by post-hoc test

**Table 4:** Analysis of the subscales of the questionnaire in the studied groups (*n* = 100)

Questionnaire’s items	Kt/V Groups	N	Mean Rank	*P* value*		*P* value**
Symptoms / problems list	Group-A	24	77.08	< 0.0001	Group-A vs Group-B	0.004
	Group-B	54	54.57		Group-A vs Group-C	< 0.0001
	Group-C	22	11.5		Group-B vs Group-C	< 0.0001
Effect of kidney disease	Group-A	24	81.9	< 0.0001	Group-A vs Group-B	< 0.0001
	Group-B	54	52.31		Group-A vs Group-C	< 0.0001
	Group-C	22	11.8		Group-B vs Group-C	< 0.0001
Burden of kidney disease	Group-A	24	71.94	< 0.0001	Group-A vs Group-B	0.095
	Group-B	54	56.86		Group-A vs Group-C	< 0.0001
	Group-C	22	11.5		Group-B vs Group-C	< 0.0001
Work Status	Group-A	24	44.25	0.003	Group-A vs Group-B	1.000
	Group-B	54	46.61		Group-A vs Group-C	0.086
	Group-C	22	66.86		Group-B vs Group-C	1.000
Cognitive Function	Group-A	24	70.19	< 0.0001	Group-A vs Group-B	0.122
	Group-B	54	56.06		Group-A vs Group-C	< 0.0001
	Group-C	22	15.39		Group-B vs Group-C	< 0.0001
Quality of Social interaction	Group-A	24	40.27	< 0.0001	Group-A vs Group-B	< 0.0001
	Group-B	54	69.23		Group-A vs Group-C	0.011
	Group-C	22	15.68		Group-B vs Group-C	< 0.0001
Sleep	Group-A	24	79.35	< 0.0001	Group-A vs Group-B	0.001
	Group-B	54	53.56		Group-A vs Group-C	< 0.0001
	Group-C	22	11.5		Group-B vs Group-C	< 0.0001
Overall Health	Group-A	24	69.88	< 0.0001	Group-A vs Group-B	0.222
	Group-B	54	57.78		Group-A vs Group-C	< 0.0001
	Group-C	22	11.5		Group-B vs Group-C	< 0.0001
1.1.Patient Satisfaction	Group-A	24	58.88	0.001	Group-A vs Group-B	0.021
	Group-B	54	41.43		Group-A vs Group-C	1.000
	Group-C	22	63.64		Group-B vs Group-C	0.003
Physical Function	Group-A	24	66.23	< 0.0001	Group-A vs Group-B	0.071
	Group-B	54	57.45		Group-A vs Group-C	< 0.0001
	Group-C	22	16.27		Group-B vs Group-C	< 0.0001
Role limitation due to Physical function	Group-A	24	56.96	0.39	Group-A vs Group-B	
	Group-B	54	50.55		Group-A vs Group-C	
	Group-C	22	43.34		Group-B vs Group-C	
General Health	Group-A	24	75.79	< 0.0001	Group-A vs Group-B	< 0.0001
	Group-B	54	37.89		Group-A vs Group-C	0.001
	Group-C	22	53.86		Group-B vs Group-C	0.018
Pain	Group-A	24	74.75	< 0.0001	Group-A vs Group-B	0.011
	Group-B	54	55.45		Group-A vs Group-C	< 0.0001
	Group-C	22	11.6		Group-B vs Group-C	< 0.0001
Emotional Well Being	Group-A	24	70.92	0.001	Group-A vs Group-B	0.171
	Group-B	54	57.31		Group-A vs Group-C	< 0.0001
	Group-C	22	11.5		Group-B vs Group-C	< 0.0001
1.1.Role limitations due to emotional function	Group-A	24	64.88	0.26	Group-A vs Group-B	
	Group-B	54	53.96		Group-A vs Group-C	
	Group-C	22	26.32		Group-B vs Group-C	
Social Functions	Group-A	24	80.35	< 0.0001	Group-A vs Group-B	< 0.0001
	Group-B	54	52.58		Group-A vs Group-C	< 0.0001
	Group-C	22	12.82		Group-B vs Group-C	< 0.0001
Energy	Group-A	24	74.5	< 0.0001	Group-A vs Group-B	0.042
	Group-B	54	55.72		Group-A vs Group-C	< 0.0001
	Group-C	22	11.5		Group-B vs Group-C	< 0.0001
Physical Composite	Group-A	24	65.92	0.002	Group-A vs Group-B	0.042
	Group-B	54	51.33		Group-A vs Group-C	0.001
	Group-C	22	31.64		Group-B vs Group-C	0.129
Mental Composite	Group-A	24	70.54	< 0.0001	Group-A vs Group-B	0.149
	Group-B	54	57.31		Group-A vs Group-C	< 0.0001
	Group-C	22	11.91		Group-B vs Group-C	< 0.0001

*P value is calculated by Kruskal-Wallis H test

**P value is calculated by Bonferroni post-hoc test

A significant difference was found regarding pre-session weight and interdialytic weight gain after improvement of kt/v in group C. (Table 5). There was a statistically significant difference regarding both dialysis session duration and PTH; with lower PTH values and longer dialysis sessions duration in patients with improved Kt/v (Table 6). Post dialysis urea, phosphorous and hemoglobin were significantly changed in group C after improvement of kt/v (Table 7).

**Table 5:** Comparison between patient general data of group C before and after improvement of kt/v

		N	Mean ± SD	Median (IQR)	*p value
Pre-session weight (kg)	Before	22	79.09 ± 13.99	79.00 (66.75–87.25)	0.031
	After		78.77 ± 14.02	79.00 (66.50–87.25)	
Post-session weight (kg)	Before	22	75.82 ± 13.91	76.00 (64.00–85.00))	0.104
	After		75.91 ± 13.99	76.00 (64.00–85.37)	
Interdialytic weight gain (Kg)	Before	22	3.21 ± 0.59	3.00 (3.00–4.00)	0.025
	After		2.86 ± 0.58	3.00 (2.5–3.00)	

*P value is calculated by paired t test

**Table 6:** Paired comparison of dialysis duration and parathyroid hormone in group C before and after improvement of kt/v

	N	Median (IQR)		*P value
		Before	After	
Dialysis session duration (hrs)	22	3.00 (3–3)	4.00	< 0.001
PTH (pg/dl) #	22	784.50 (343.50–979.50)	632.00(343.50–873.75)	0.002

*P value was computed by Wilcoxon Signed Ranks test

**Table 7:** comparison between patient laboratory data of group C before and after improvement of kt/v:(*n* = 22)

		N	Mean ± SD	Median (IQR)	*p* value*
Pre-session Urea (mg/dl)	Before	22	129.0 ± 35.17	128.50 (98.75–156.00)	0.668
	After		132.00 ± 20.43	133.00 (121.25–147.00)	
Post-session Urea (mg/dl)	Before	22	56.36 ± 17.32	59.50 (40.00–68.50)	0.001
	After		43.73 ± 6.73	46.50 (39.75–48.25)	
Serum calcium (mg/dl)	Before	22	8.93 ± 0.88	9.00 (8.33–9.45)	0.322
	After		8.75 ± 0.79	8.9 (8.03–9.43)	
Serum phosphorus (mg/dl)	Before	22	5.37 ± 0.75	5.40 (4.88–6.03)	< 0001
	After	22	4.06 ± 0.59	4.00 (3.75–4.50)	
Serum albumin (g/dl)	Before	22	3.28 ± 0.46	3.20 (2.98–3.50)	0.329
	After		3.23 ± 0.49	3.15 (2.88–3.43)	
Hemoglobin (g/dl)	Before	22	9.17 ± 0.78	9.10 (8.50–9.73)	< 0001
	After		10.90 ± 0.88	10.00 (9.50–10.80)	
Kt/v	Before	22	0.99 ± 0.12	1.03 (0.96–1.08)	< 0001
	After		1.31 ± 0.06	1.32 (1.25–1.35)	

*P value is calculated by paired t test

All the studied subscales showed significant change after improvement of kt/v except for Work status, Patient satisfaction, Role limitation for physical and Role limitation due to emotional problems (Table 8).

**Table 8:** Analysis of the subscales of the questionnaire in group C before and after improvement of kt/v

Subscales of the questionnaire	N	Median(IQR)		***P*** value*
		Before	After	
Symptoms / problems list	22	39.58 (35.42–45.33)	71.88 (41.67–79.17)	< 0.001
Effect of kidney disease	22	50.00 (46.09–53.13)	59.38 (50.00–68.75)	0.003
Burden of kidney disease	22	31.25 (25.00–31.25)	50.00 (31.25–62.50)	0.001
Work status	22	50.00 (50.00–50.00)	50.00 (0.00–50.00)	0.248
Cognitive function	22	73.33 (66.67–73.33)	80.00 (71.67–86.67)	0.003
Quality of social interaction	22	46.67 (38.33–53.33)	66.67 (40.00–73.33)	0.002
Sleep	22	35.00 (27.50–40.63)	62.50 (38.13–70.00)	0.001
Overall health	22	30.00 (20–30)	70.00 (30.00–70.00)	0.001
Patient satisfaction	22	50.00 (45.83–54.17)	41.66 (33.33–50.00)	0.115
Physical function	22	32.50 (25.00–35.00)	45.00 (33.75–55.00)	0.002
Role limitation due to physical function	22	10.00 (0.00–50.00)	25.00 (25.00–50.00)	0.415
General health	22	55.00 (45.00–11.25)	47.50 (40.00–55.00)	0.060
Pain	22	12.50 (10.00–22.50)	45.00(20.00–55.63)	0.001
Emotion well-Being	22	38.00 (32.00–44.00)	60.00 (40.00–68.00)	0.001
Role limitations due to emotional problems	22	33.33 (0.00–33.33)	33.33 (25.00–66.67)	0.194
Social function	22	25.00 (12.50–37.50)	50.00 (25.00–62.5.00)	0.002
Energy	22	25.00 (20.00–26.50)	45.00 (23.75–55.00)	0.001
Physical composite	22	32.49 (29.30–36.90)	36.55 (33.99–41.22)	0.042
Mental composite	22	28.05 (24.66–32.21)	35.40 (27.49–46.86)	0.002

*P value was computed by by Wilcoxon Signed Ranks test

Regarding linear study of Kt/v and the summarizing domains of SF-36 (physical and mental composites), the correlation of Kt/v and these domains controlled for two important correlated confounders, namely Hemoglobin and PTH showed statistically significant positive correlation. When Work status was correlated to Kt/v (controlled also to PTH, hemoglobin), it didn’t show any significance (*r* = − 0.168, *p* = 0.068) (Table 9).

**Table 9:** Partial correlation of Kt/v with some subscales of KDQOL-SF™ questionnaire (Control variables are PTH, hemoglobin)

	Work status	Physical composite	Mental composite
Kt/v r	−0.168	0.304	0.587
p	0.068	0.002	< 0.0001

## Discussion

ESRD is an important problem gradually increasing worldwide [1]. It has a considerable impact on the functional status and quality of life (QOL) perceived by the patient. Even in relatively early stages of chronic kidney disease, it may be mainly due to accumulation of uremic toxins which result in uremic symptoms such as dry skin, sleep difficulty, itching, numbness/tingling, decreased interest in sex, and bone/joint pain which may interrupt daily-life activities of patients [19]. HD is the most commonly used treatment option for this stage [2] a method that removes large amounts of uremic toxins with expected improvement of quality of life. However, hemodialysis, in itself, can lead to significant changes in patients’ lifestyle and may possibly impair its quality [3]. This may be due to hemodialysis related complications as muscle cramps, pruritus, anorexia and access problems.

The kidney Disease Outcomes Quality Initiative (K/DOQI) recommended monitoring the Health Related Quality of Life (HRQOL) for all patients with renal disease [20]. Although there are several standard questionnaires available for assessment of quality of life, the KDQOL-SF is the most commonly used one. It has many advantages compared to other instruments; it has been tested in several populations with kidney disease, has both general and specific modules to assess chronic kidney disease, can be used both for patients on HD and peritoneal dialysis, has questions about the sexual area, and can be self-applied or applied by an interviewer [13]. In a multitude of research, a strong correlation between HD dose and clinical outcomes has been described, and dialysis adequacy is now considered a strong predictor for morbidity and mortality of ESRD patients treated with regular hemodialysis [21]. Dialysis adequacy and optimal dosing can be assessed utilizing several techniques and methods; among which, the Kt/v determined by single pool urea kinetic modeling, is the most frequently used and preferred method for the numerical expression of dialysis dose and or adequacy, as it is more specific and accurate [22]. A minimum Kt/V units per dialysis session of 1.2, carried out 3 times per week, has been recommended as an acceptable target; a recommendation that was essentially advocated by the well-conducted HEMO study [23].

In Egypt, especially in our locality, many physicians looking after hemodialysis patients are interested mainly in the clinical and laboratory data of their patients, paying little attention to the patients’ emotional satisfaction and their quality of life. The present study was planned with a main focus of assessment of HRQOL among ESRD patients treated with hemodialysis in relation to different variables, especially the adequacy of dialysis relying on the calculated kt/V.

So, this work was carried out on 100 ESRD patients (51% males; with mean age of 48 ± 5.89), maintained on regular hemodialysis three times weekly. Dialysis adequacy was assessed by single pool Kt/V, and KDQOL-SF questionnaire was utilized for determining the quality of life. Taking KDIGO (2012) guidelines for anemia in ESRD in consideration, nearly half of patients in the current study fulfilled the target hemoglobin [24], while 45% of the patients were within the recommended standard levels of serum calcium according to KDIGO (2009) guidelines and one fifth suffered from low serum calcium [25]. Regarding serum phosphorus, utilizing the recommended values of KDIGO guidelines, nearly a quarter of the patients had hyperphosphatemia, while hypophosphatemia was encountered less frequent. On the other hand, more than one third of the patients had hyperparathyroidism by the previous reference criteria. Hypoalbuminemia afflicted many patients in the present study; this might be due to anorexia, malnutrition and inflammation which are common findings in HD patients [26, 27].

The baseline Kt/v of the majority of patients in the current study, with a mean value of 1.4, was within the target recommendation by the National kidney foundation Disease Outcomes Quality Initiative [20]clinical practice guidelines, > 1.2, which reflects a reasonable patient care service. However, recent guidelines recommended higher value > 1.4 which reflect more interest of the international community in improving the dose of dialysis that could have positive impacts on patients quality of life [28].

The physical domain includes three subscales; namely, physical function, role limitation due to physical function, and bodily pain. In previous studies this domain was reported to be a significantly associated predictor of both death and hospitalization [29, 30]. This argues for the importance of improving the physical component in hemodialysis patients; a change that is expected to effectively decrease the risk of death and recurrent hospitalization for these patients with less cost on the community [30].

In the current study each of the three subscales of the physical domain had a score of below an average of 50, suggesting that these patients are suffering from poor physical performance. This is in agreement with the corresponding findings reported for the European and American patients in DOPPS study [31]. However, in the latter study the Japanese patients had a score which was above the previously mentioned average, which are thought to reflect their better awareness of the clinical problems and possibly denoting better health education conducted by their caring staff.

Many studies have proposed several points for improving the component of the physical domain. Physical exercise has been the focus of interest in a good body of literature [32–34]. In a systematic review of 29 clinical trials, improvement of the physical condition following the use of aerobic training was observed [35]. The importance of physical exercise not only has a valuable effect on the physical component of QOL, but also improves cardiopulmonary fitness, anemia, hyperlipidemia, chronic inflammation, blood pressure, insulin resistance, anxiety, depression and adequacy of dialysis [36, 37]. Although the physical exercise in hemodialysis patients has many beneficial effects and it should be mandatory not optional for patients with ESRD, exercise programs are still not a part of routine clinical practice in many countries [38].

On the other hand, the mental domain includes social function, role limitation due to mental function and general mental health. The first two subscales, in the present study, scored above 50 while the third one (mental health) approached 100 which represents the maximum score for this subscale. These findings are in accordance with the corresponding findings of Czyzewski.et.al., 2014 and the DOPPS study [17, 30]. The mental status of the hemodialysis patients is intimately linked to the social status which is expected to be badly changed by their illness and inability to work and get suitable financial coverage. These patients might consequently lose their self-esteem and feel handicapped. Moreover, hemodialysis itself may cause mood and sleep disorders which would result in more mental suffering, imposing much burden on the caring nephrologists and dialysis staff.

Important points to improve mental status should include training of patients to talk with their doctors whenever they feel down, anxious, worried, nervous or fearful. This may help to assess their problems and start to plan for solutions. Group therapy is another successful way that can improve mental health as not only has a self-confidence effect, but also it enables the patients to feel that they are not alone in their struggle with dialysis [39]. Other ways of intervention may include using antidepressant drugs which should be prescribed in the proper modified doses by caring physicians [40]. More ideas in this respect may comprise utilizing entertainment tools in dialysis wards, such as televisions, radio and magazines, recreational group outings, and frequently improving the decoration of dialysis rooms.

In addition to the above-mentioned subscales within the mental and physical domains, two other subscales, namely, energy and general health perception are expected to be reflected on both of them. In the current study, both energy and general health perception showed an average or even below average scores; findings that are in parallel with those reported in a study by Merkus and colleagues, 2017 [41].

Kt/v, determined by single pool urea kinetic modeling, is the most frequently used and preferred method for the numerical expression of dialysis dose and or adequacy, as it is more specific and accurate [42]. In the current study Kt/V was utilized to divide the patients into three groups of highly adequate, intermediately adequate and inadequate, for the purpose of analytical comparisons, relying on a Kt/V cut points of 1.2 and 1.5. A similar way of subdividing the patients relying on cut-off values of Kt/V was also adopted by many previous groups of researches. Akhil Babu et al. [43], divided his studied patients into two groups of adequate and inadequate dialysis efficiency, utilizing a Kt/V value of 1.4 as a separator. While two other studies [44, 45] divided their patients according to a Kt/V of < 1.2 and ≥ 1.2.

In the present work, the group with highest Kt/V was noticed to have a mean dry weight which is higher than that of the other two groups; an explanation of that may be related to their better dialysis adequacy leading to lower GIT problems, better appetite, lower inflammation and higher anabolism [46]. However, following improvement of the dialysis adequacy for 3 months, the mean dry weight did not show significant changes. Lack of putting on weight in this situation could probably reflect insufficient period of observation; had they been given more time with the better Kt/V could have been translated into better body building and more weight gain.

Adequacy of dialysis is related to many factors; for example, duration and frequency of dialysis sessions, dialyzer size and its characteristics, dialysate and blood flow rates, and nature of vascular accesses and whether or not blood recirculation existed [47]. In the current study, the group of poorest dialysis adequacy was noticed to ask for termination of their sessions ahead of time. This non- compliance in sticking to the proper session duration was usually also associated with low compliance regarding diet, salt and water intake, which could be an explanation for their higher interdialytic weight gain in this particular group in comparison to the other two groups. Consequently, increasing the duration of sessions was utilized to improve the dialysis adequacy in this group. Alongside with the significant increase in session duration, there was significant consequent decrease in the interdialytic weight gain.

Having appreciated the positive impact of improving dialysis adequacy on many clinical and biochemical data of patients [6, 48], one would expect welcome effects on the quality of life. In the present research, the quality of life variables were compared among the three groups. These variables were also compared before and after improving dialysis adequacy in group C. All the subscales of the physical domain, except role limitations due to physical function impairment, were found to be significantly low in group C. These low scores were parallel to results reported in similar studies [49, 50]. Work status was significantly higher in group C (*p* = 0.003 but post hoc analysis didn’t show significance between groups). When Work status was correlated to Kt/v (controlled to PTH, hemoglobin), it didn’t show any significance (*r* = − 0.168, *p* = 0.068). We were not astonished by this non- significant correlation as this subscale was already higher (better) in group C (lowest Kt/v) than the other 2 groups and improving Kt/v wouldn’t render a significant change in this group.

The physical domain subscales, apart from the subscales concerning the general health, role limitations due to physical function impairment and work status, were improved after improving of Kt/V. This improvement in the majority of subscales of the physical domain was associated with improvement in the physical composite score by 12.5%; as this latter score is calculated from all of the above mentioned subscales.

Likewise, all the subscales of the mental and kidney domains were significantly lower in the same group of poorest adequacy; findings confirming previously published data [49, 50], some of these subscales did not even show improvement after ameliorating the dialysis adequacy. It is worthwhile to note that the improvement in the mental health composite was two-fold higher than that in the physical health composite. This preferential improvement may possibly be ascribed to repeated patient-doctor interactions during the study which could raise the interest in the concept of quality of life among the patients. This might promote the doctors to pay more attention towards their patients’ psychological aspect during reassessment of the KDQol-SF.

One possible explanation for lack of improvement of some subscales after enhancement of the dialysis dose, in spite of the fact that they were better in group A (Kt/v > 1.5), could be their possible need for more time to achieve their fully expressed response.

Many previous studies examined the association between adequacy of dialysis mostly using Kt/V and the HRQOL; some showed no significant association but others showed dissimilar results [4, 41, 51]. These results are in contrast with the result in the current study; differences might be due to the small size sample [51], collection of data just after 3 months from the start of dialysis – a time when residual renal function may still be present and a duration that may be too short to have an effect on the quality of life [41]. Another difference could be using urea reduction rate as a single assessment tool for adequacy of dialysis [4].

The present study has the merit of comparing three groups of patients based on kt/v, one of them above the target and another below the lower target of 1.2, which allowed scrutiny the effects of kt/ v on different subscales of Qol. Another good point is the paired comparisons of the Qol variables before and after enhancing dialysis adequacy which could envision the benefits of improving the kt/v on the patients’ perception of their Qol.

Having said that, the present research has got some limitations; firstly, the duration of follow up after improvement of kt / v was limited to 3 month – a duration that might not permit full expression of improvement of the quality of life especially in the physical aspect. Secondly, the present study has relied on single measurements of kt/v which might not be as accurate indicator of dialysis adequacy as multiple measurements of the same tool over the whole period of observation.

## Conclusion

One would suggest that hemodialysis service providers should have a continuous quality of life assessment plan and make corrective actions when necessary to improve it. Detection and improvement of suboptimal dialysis adequacy is one important point in this aspect as this would have a welcome effect on patients’ capabilities as productive members of community.

## Data Availability

All data analyzed during this study are included in this manuscript.
